# Tunnelled haemodialysis catheters in central Free State: Epidemiology and complications

**DOI:** 10.4102/sajr.v23i1.1791

**Published:** 2019-11-27

**Authors:** John Fox, Gina Joubert, Eugene Loggenberg

**Affiliations:** 1 Department of Radiology, Universitas Academic Hospital complex, University of the Free State, Bloemfontein, South Africa; 2 Department of Biostatistics, University of the Free State, Bloemfontein, South Africa

**Keywords:** End-stage renal disease, tunnelled haemodialysis catheters, epidemiological profile, complication rate

## Abstract

**Background:**

End-stage renal disease (ESRD) is a disease with profound impact on the patient, health system and economy. Tunnelled haemodialysis catheters (TDC) are amongst the most common dialysis methods. It has been established internationally that certain demographic descriptors and aetiologies carry an increased risk of complications. There is a dearth of epidemiological profiling of ESRD patients with TDC in South Africa.

**Objective:**

To establish the epidemiological profile of patients who received TDC and to establish the complication rate, with the goal of demonstrating associations between the epidemiological profiles and complications.

**Method:**

This was a retrospective study of all patients who received TDC in an Academic Hospital Interventional Radiological Suite over a period of 60 months between 01 March 2011 and 29 February 2016.

**Results:**

A total of 179 patients received 231 catheters. The majority of patients were male (58.7%) and 35.8% of the patients resided in Mangaung. The leading aetiologies of ESRD included hypertensive nephropathy (43.6%), primary glomerular disease (17.3%) and HIV-associated nephropathy (6.1%). Procedural complications occurred in 7/224 (3.1%) insertions, whilst 37/185 (20.0%) developed catheter-related infection and 54/185 (29.2%) developed dysfunctional catheters. There were no deaths linked to catheter-related complications.

**Conclusion:**

Our patients’ demographic profile, ESRD aetiology, complication rate for procedural complications and catheter-related infections are roughly on par with international studies; however, the catheter dysfunction rate is higher than in the aforementioned studies. This possibly reflects the difficulty of accessing specialist care for our patients, the majority of whom reside outside the Mangaung district. Further studies with larger sample sizes are required to demonstrate statistically relevant associations.

## Introduction

End-stage renal disease (ESRD) is an increasing healthcare concern across the world with a high mortality rate and associated economic implications, particularly in Southern Africa, where it affects a younger demographic than in developed countries.^1,2^ An effective screening programme would assist in early nephrologist or renal centre referral which is shown to have an impact on decreasing the morbidity and mortality of these patients.^3,4^

In state healthcare, 44.1% of the dialysis population is managed with haemodialysis and at our institution a large portion of the dialysis population undergo tunnelled haemodialysis catheter insertion either for temporary vascular access (whilst grafts or fistulae mature or the peritoneum recovers) or when other vascular access routes are exhausted.^5^ Tunnelled haemodialysis catheters (TDC) do offer some advantages, including immediate dialysis and no repeated venepuncture. However, they are associated with an increased risk of complications and significant mortality when compared with other types of vascular access, with a 1-year survival of patients on TDC of 75%.^3,6^

Based on studies in China and Croatia, multiple risk factors have been demonstrated to carry an increased risk of complications.^3,7^ However, no local study has assessed our complication rate and investigated epidemiological risk factors. Filling this void would assist in the implementation of focused and effective screening programmes.

The goal of this study was multifactorial. We aimed to establish the epidemiological profile of patients at an academic hospital, who received TDCs at the Interventional Radiological Unit over a 60-month period, to establish the complication rate within that population group and to determine if associations between the risk factors, epidemiological data and complications could be established.

## Research methods and design

### Study design and setting

This was a retrospective, analytic study conducted at an Academic Hospital Interventional Radiology Unit, which serves the population of the Free State province, as well as occasional out of province and private patients.

### Study population and sampling strategy

The study population consisted of all state patients who received TDCs at an Academic Hospital Interventional Radiology Unit during the period of 01 March 2011 to 29 February 2016. All patients aged 18 years and older, who received their catheter at the interventional suite, were included.

### Catheter insertion

Catheters were inserted by an experienced interventional radiologist in the Interventional Unit via percutaneous access. The procedure was performed under sterile theatre conditions with ultrasound guided venous access. All TDCs inserted in our centre are cuffed. The catheter is tunnelled subcutaneously for approximately 9 cm – 10 cm from the venous access site. The catheter is then placed under fluoroscopic control with tip positioning in the right atrium. Cutaneous fixation is created with sutures until cuff adhesion – approximately 8–12 weeks. Initial patency and positioning are confirmed during the procedure. The catheter is then locked with heparin (1000 μ/mL)

The primary goal for access was the internal jugular vein. However, in patients with previous access and complications, other sites were used. Subclavian access was used when no other access site was available.

### Secondary intervention

In patients where the catheter is unable to maintain adequate extracorporeal blood flow and thrombolytic therapy (alteplase) has been ineffective, brushing is performed in the Interventional Unit under fluoroscopic guidance and sterile conditions to displace and remove the fibrin sheath (a composite of cells and debris that forms a biofilm around catheters that can obstruct the lumen, acting as a valve) or thrombus by using a Terumo guidewire to sound the catheter lumen and rinse the lumen with saline. The catheter is then locked with heparin, 1000 μ/mL. If brushing fails to restore patency, then snaring is employed – vascular access is gained from another site and mechanical stripping of the catheter tip is performed via a snare.

### Data collection

Patients were identified using the procedural register and further information was gathered from existing electronic medical records. A comprehensive data sheet was completed. Details captured included date of birth, age at catheter insertion and residence. Aetiology was grouped into diabetes, primary glomerular disease (including nephrotic syndrome, acute glomerulonephritis and rapidly progressive glomerulonephritis), hypertensive nephropathy, acute renal failure, obstructive uropathy, renal tubular interstitial diseases (including acute tubular necrosis, tubulointerstitial nephritis, contrast nephropathy, reflux nephropathy and myeloma), Human Immunodeficiency Virus Associated Nephropathy (HIVAN), drug induced nephropathy, polycystic kidney disease and unknown.

For ease of analysis, complications were grouped into procedural complications (air embolism, bleeding and pneumothorax), catheter-related infection and catheter dysfunction (malposition, thrombosis, fibrin sheath, central vein stenosis and loosening or catheter breakage).

Further details recorded included whether the catheters underwent repair or brushing and if they were removed because of complications, fistula maturation or peritoneal dialysis catheter use. In the cases of patient demise, it was noted whether this was a result of catheter-related complications or other causes.

Primary and secondary patency was calculated. Primary patency is regarded as the time duration of catheter patency until the first intervention required to maintain patency whilst secondary patency is regarded as the length of time from insertion until catheter removal because of complication or catheter failure.^8^

### Data analysis

The primary researcher entered all the data onto an Excel data sheet, which was then submitted for statistical analysis by the Department of Biostatistics at the University. Results were summarised as frequencies and percentages (categorical variables) and means, standard deviations and percentiles (numerical variables). Associations were investigated using appropriate hypothesis testing with *p* <0.05 considered statistically significant.

### Ethical considerations

Ethical clearance was obtained from the Health Sciences Research Ethics Committee of University of Free State (HSREC 62/2017) and Free State Department of Health (UFS-HSD2017/0478).

## Results

A total of 179 patients received TDCs during the study period and qualified for the study. In the study sample, 105 were male (58.7%) and 64 (35.8%) resided in Mangaung district. The mean age at insertion was 40.4 years with a standard deviation of 12.05. The four leading aetiologies were hypertensive nephropathy, primary glomerular disease, HIVAN and unknown aetiology (see Table 1 for more information).

**TABLE 1:** Aetiology per patients (*n* = 179).

Aetiology	*n*	%
Diabetes	7	3.9
Primary glomerular disease	31	17.3
Hypertensive nephropathy	78	43.6
Vasculitis	5	3.0
Acute renal failure	1	0.6
Obstructive uropathy	5	3.0
Renal tubular interstitial diseases	3	1.7
HIVAN	11	6.1
Drug induced	5	3.0
Polycystic kidney disease	8	4.5
Unknown	34	19.0
**Other**	
HELLP	1	0.6
Lupus nephritis	2	1.1
Nephrectomy due to malignancy	1	0.6
Oligomegaphronia	1	0.6
Oncocytoma	1	0.6

HELLP, haemolysis, elevated liver enzymes, low platelet count syndrome.

The patients received 231 catheters. A hundred and fifty-eight patients had catheters inserted for the first time. The majority of patients (141, 77.3%) received one catheter, 25 patients (14.0%) received two, 10 patients (5.6%) received three, 1 patient (0.6%) received four and 1 patient (0.6%) received five catheters during the study period. Of the 231 catheters inserted, 224 (97.0%) had information regarding insertion and 185 (80.1%) had information regarding follow up. The majority of lines were inserted in the right internal jugular vein, with the left internal jugular vein insertion being the second as per Table 2.

**TABLE 2:** Site of insertion (*n* = 224).

Site of insertion	*n*	%
Left internal jugular	22	9.8
Left common femoral	13	5.8
Left subclavian	2	0.9
Right internal jugular	165	73.6
Right femoral	14	6.2
Right subclavian	8	3.6

Note: Sites of insertion were recorded for 224 of the 231 catheters inserted.

Procedural complications occurred in 3.1% of insertions whilst 20.0% developed catheter-related infections and 29.2% developed complications related to dysfunction (see Table 3 for further breakdown).

**TABLE 3:** Complications and incidence.

Complications	*n*†	%
**Procedural (*N* = 224)**		
Air embolism	1	0.4
Bleeding	7	3.1
Pneumothorax	0	0
**Catheter dysfunction (*N* = 185)**		
Thrombosis	25	13.5
Fibrin sheath	20	10.8
Central vein stenosis	5	2.7
Catheter loosened	9	4.9
Dysfunction due to malpositioning	4	2.2
**Catheter-related infection (*N* = 185)**		
Catheter-related infection	37	20.0

Note: Procedural complication was recorded during initial catheter insertion and admission and thus has a larger denominator than catheter-related infection and dysfunctional complications which were recorded in patients who returned for follow up. More than one complication could occur per insertion.

† , Denominators are procedures.

The mean age at insertion varied between the complication groups: in the catheter-related infection group, the mean age was 37.5 years; in the procedural complication group, mean age was 40.2 years; and in the catheter dysfunction group, mean age was 39.8 years. Table 4 summarises the patient characteristics, complications recorded and the associations between them.

**TABLE 4:** Complications and associations per catheters.

Characteristic	Procedural complications† and catheters: 7/224		*p*	Catheter-related infection and catheters: 37/185		*p*	Dysfunctional complications and catheters: 54/185		*p*
	*n*	%		*n*	%		*n*	%	
**Gender**	-	-	0.7	-	-	0.16	-	-	0.35
Male	3/126	2.4		16/99	16.2	-	26/99	26.3	-
Female	4/98	4.1		21/86	24.4	-	28/86	32.6	-
**Residing**	-	-	0.69	-	-	0.65	-	-	0.74
Mangaung	3/77	2.7	-	12/66	18.2	-	18/66	27.3	-
Outside district	4/127	3.9	-	25/119	0	-	36/119	30.3	-
**Aetiology**‡	-	-	-	-	-	-	-	-	-
Diabetes	0/9	-	-	43472	14.3	-	43503	28.6	-
Primary glomerular disease	1/39	2.6	-	11/33	33.3	-	7/33	21.2	-
Hypertensive nephropathy	4/94	4.3	-	10/77	12.9	-	21/77	27.3	-
Renal tubular interstitial disease	0/6	-	-	2/6	33.3	-	1/6	16.7	-
HIVAN	0/12	-	-	3/9	33.3	-	2/9	22.2	-
Polycystic kidney	0/12	-	-	2/10	20.0	-	7/10	70.0	-
**Site of insertion**	-	-	0.38	-	-	0.01	-	-	0.38
Left femoral	1/13	7.7	-	4/11	36.4	-	5/11	45.5	-
Left subclavian	0/2	-	-	1/2	50.0	-	1/2	50.0	-
Left internal jugular	1/22	4.6	-	4/22	18.2	-	9/22	40.9	-
Right femoral	1/14	7.1	-	7/12	58.3	-	4/12	33.3	-
Right internal jugular	4/165	2.4	-	20/132	15.2	-	34/132	25.8	-
Right subclavian	0/8	-	-	1/6	16.7	-	1/6	16.7	-

† , Procedural complications were recorded during initial catheter insertion and admission and thus consist of a larger pool than catheter-related infection and dysfunctional complications which were recorded in patients who returned for follow up; ‡, Only aetiologies with five patients or more were included in this table.

Out of the 231 catheters, 45 catheters (19.5%) had incomplete follow up. Of the catheters with adequate follow up, 4.3% went on to receive catheter repair, 17.7% required a single brushing, 5.4% received two brushings and 3.2% received three brushings, with a primary patency rate of 98 days. Complications resulted in 27.9% of the catheters being removed whilst 32.3% were removed because of fistulas and 18.8% because of peritoneal dialysis being initiated or resumed. No patients demised because of catheter-related complications, whilst 10.2% of the patients demised because of other causes. Secondary patency rate was 87.0% at 6 months and 76.1% at 12 months.

## Discussion

The high financial burden of ESRD has a considerable impact on the limited resources of the South African health system. Therefore, it would be of benefit if there was earlier diagnosis and efficient management of renal disease, preventing or delaying the progression to ESRD. The Academic Hospital Interventional Radiology Unit assists with TDC insertion for a large percentage of the Free State dialysis population as it can be demonstrated by considering that in 2016 the Free State had 235 patients on dialysis; our study population over the five year period numbered 179 patients.^5^ Despite the increased risk of infection and mortality compared with fistulae or grafts, TDCs remain an important part of dialysis patient care.^9,10,11^

The epidemiological analysis of the study population revealed that the patient’s age (mean of 40.4 years) was in keeping with a local South African study on ESRD, but younger than studies from other African countries and developed countries where renal failure is predominantly a diagnosis of the middle aged and the elderly.^1,2^ Male patients formed 58.7% of the sample; this corresponds to previously reported rates in Africa of 61% – 63% male gender in renal failure patients.^1^ The female proportion of the study population experienced the majority of the complications, however, the gender discrepancy was not found to be statistically significant, which is also in keeping with an international study which indicated that patient gender did not impact catheter survival.^12^

A significant percentage (64.2%) of the study population resided outside the Mangaung district with implications in terms of ease of access to specialised medical services and further management of the TDC and the patient. The patients outside the Mangaung district experienced the majority of the complications (57% – 68%) across all three complication groups although the discrepancies were not statistically significant.

End-stage renal disease aetiology was similar to other studies in Africa with hypertension being the most commonly recorded cause in 43.6% of patients versus 34.6% (Sudan) and 30.9% (Cameroon). Further common causes in our study included primary glomerular disease and HIVAN. In Cameroon, other aetiologies included glomerulonephritis (15.8%), diabetes (15.9%), HIVAN (6.6%) and unknown (14.7%).^13^ In a Sudanese study the causes included chronic glomerulonephritis (17.6%), diabetes (12.8%) obstructive uropathy (9.6%) and in 10.7% no cause was identified.^1^

Hypertension as an aetiology constituted a larger percentage of this study population than international studies although it is difficult to determine whether this was primary hypertension or secondary to chronic kidney disease. Additionally, this study had a high percentage of patients with an unknown cause. These findings could be a reflection on the lack of efficient primary healthcare with many patients presenting late in the course of the disease and not receiving renal biopsies.

The majority of catheters were inserted in the right jugular vein, with no statistically significant discrepancy between site of insertion and procedural or dysfunctional complication rate, however, there was a statistically significant correlation between catheter-related infection and insertion of the catheter in either femoral site. In a study by Dewelter et al, it was demonstrated that right jugular insertion confers a significantly improved outcome as compared with other sites of insertion.^14^

This study, as compared with a study in Pakistan, had a decreased incidence of procedure-related complications (3.2% vs. 5.6%) but an increased rate of catheter-related infection (20% vs. 17.3%) as well as dysfunction-related complications (29.2% vs. 16%).^15^ The increased incidence of catheter-related infection and complications causing dysfunction reflect perhaps the difficulty for our patients in accessing specialist care after the procedure, particularly if they reside in another district. In light of the above, it might be of value to consider a chronic low dose of aspirin to maintain tunnelled central venous catheter (CVC) patency.^16^

Catheter-related infections remain a significant problem within the dialysis population with implications for cost of care and patient quality of life, as patients with catheter-related infections have an average hospital stay of 6.5 days, undergo several tests and receive treatment during the hospital stay.^17^ Considering the incidence of catheter-related infections, future studies could analyse the benefit of antimicrobial barrier caps in reducing this rate, as per the Kidney Disease Outcomes quality Initiative (KDOQI) guidelines from the National Kidney Foundation.^16^

The secondary patency rate is better than a study in India at 6 months (87.0% compared with 55%) and the 12-month catheter survival rate falls within the wide range found in a previous review article of 2007 (between 25% – 75%).^8,18^ A high percentage of the catheters were removed because of initiating or resuming peritoneal dialysis or use of fistulae. This is perhaps because of the increased number of patients in state healthcare who are on peritoneal dialysis compared with private healthcare (27.8% vs. 6%).^5^ There were no deaths in our study because of catheter-related complications.

The aetiology in the study population, on average, did not have a statistically significant impact on the complication rate although other studies have shown that diabetes conveys increased risk and that age can have an influence additionally.^7^ Polycystic kidney disease was shown to have an increased risk of catheter-dysfunction-related complications. The reason for this is unknown and merits further investigation.

Although our study was unable to establish a statistically significant association between demographics, aetiology and complications in the majority of cases, we were, however, able to demonstrate an association between femoral site catheter insertion and the risk of catheter-related infection; and between patients with polycystic kidney disease and an increased risk of catheter dysfunction. Studies have shown that associations exist between several patient characteristics (male gender, increased age, diabetic nephropathy, hypertensive nephropathy and glomerulonephritis) and their risk of complications.^3,7^

## Study limitations

Many patients who had their catheter inserted and were then managed further in other centres were lost to follow up, resulting in incomplete information, particularly with regards to catheter-related infection and dysfunctional catheter complications. A further challenge was the relative paucity of renal biopsies to confirm the ESRD aetiology.

## Conclusion

Our demographics, aetiology of ESRD and complication profile largely correspond to other studies except for an increased complication incidence in females, an increased percentage of hypertension as the cause for ESRD and an increased percentage of catheter dysfunction complications. These findings are perhaps a reflection on the challenges our primary healthcare system faces and the difficulty for these patients to access specialist care in the periphery. Because of the limited number of patients and complications, this study was unable to establish statistically significant correlations between complications and epidemiological factors in many of the measured characteristics.

In our setting, given pre-existing research that has demonstrated a decreased risk of complications with early referral to specialist care and dialysis initiation with other vascular access options (besides TDC),^4^ it would be optimal to create a screening programme for high risk patients (HT, DM).^2^ If a South African multicentre study with a larger study population was able to confirm local risk factors for complications, then appropriate care centres could implement protocols for increased vigilance and screening for complications in the vulnerable population groups. This could also lead to and assist with the formation of local guidelines for the management of dialysis such as the KODQI 2018 guidelines.^16^ Together, these could assist in early identification of patients at risk of developing ESRD and lead to earlier referral to specialist care which has been shown to have a positive effect on patient outcome.^4,18,19,20^

## References

[1] Banaga A, Mohammed E, Siddig R, et al Causes of end stage renal failure among haemodialysis patients in Khartoum State/Sudan. BMC Res Notes. 2015;8(1):1–7. 10.1186/s13104-015-1509-x 26419536 PMC4589074

[2] Naicker S Burden of end-stage renal disease in sub-Saharan Africa. Clin Nephrol. 2010;74(Suppl. 1):S13–S16. 10.5414/CNP74S013 20979956

[3] Pašara V, Maksimović B, Gunjača M, et al Tunnelled haemodialysis catheter and haemodialysis outcomes: A retrospective cohort study in Zagreb, Croatia. BMJ Open. 2016;6(5):e009757 10.1136/bmjopen-2015-009757 PMC4874139 27188801

[4] Astor BC, Eustace JA, Powe NR, Klag MJ, Fink NE, Coresh J Type of vascular access and survival among incident hemodialysis patients: The choices for healthy outcomes in caring for ESRD (CHOICE) Study. J Am Soc Nephrol. 2005;16(5):1449–1455. 10.1681/ASN.2004090748 15788468

[5] Davids MR, Jardine T, Marais N, Jacobs JC South African Renal Registry Annual Report 2016. Afr J Nephrol. 2018;21(1):60–72. 10.21804/21-1-3298

[6] Moist L, Trpeski L, Na Y, Lok CE Increased hemodialysis catheter use in Canada and associated mortality risk: Data from the Canadian Organ Replacement Registry 2001–2004. Clin J Am Soc Nephrol. 2008;3(6):1726–1732. 10.2215/CJN.01240308 18922993 PMC2572294

[7] Wang K, Wang P, Liang X, Lu X, Liu Z Epidemiology of haemodialysis catheter complications: A survey of 865 dialysis patients from 14 haemodialysis centres in Henan province in China. BMJ Open. 2015;5(11):e007136 10.1136/bmjopen-2014-007136 PMC4663418 26589425

[8] Bagul A, Brook N, Kaushik M, Nicholson M Tunnelled catheters for the haemodialysis patient. Eur J Vasc Endovasc Surg. 2007;33(1):105–112. 10.1016/j.ejvs.2006.08.004 17067828

[9] Lok CE, Foley R Vascular access morbidity and mortality: Trends of the last decade. Clin J Am Soc Nephrol. 2013;8(7):1213–1219. 10.2215/CJN.01690213 23824198

[10] Ravani P, Palmer SC, Oliver MJ, et al Associations between hemodialysis access type and clinical outcomes: A systematic review. J Am Soc Nephrol. 2013;24(3):465–473. 10.1681/ASN.2012070643 23431075 PMC3582202

[11] Napalkov P, Felici DM, Chu LK, Jacobs JR, Begelman S Incidence of catheter-related complications in patients with central venous or hemodialysis catheters: A health care claims database analysis. BMC Cardiovasc Disord. 2013;13:86 10.1186/1471-2261-13-86 24131509 PMC4015481

[12] Fry AC, Stratton J, Farrington K, et al Factors affecting long-term survival of tunneled haemodialysis catheters – A prospective audit of 812 tunnelled catheters. Nephrol Dial Transplant. 2008;23(1):275–281. 10.1093/ndt/gfm582 17890252

[13] Halle MP, Takongue C, Kengne AP, Kaze FF, Ngu KB Epidemiological profile of patients with end stage renal disease in a referral hospital in Cameroon. BMC Nephrol. 2015;16:59 10.1186/s12882-015-0044-2 25896605 PMC4413994

[14] Develter W, De Cubber A, Van Biesen W, Vanholder R, Lameire N Survival and complications of indwelling venous catheters for permanent use in hemodialysis patients. Artif Organs. 2005;29(5):399–405. 10.1111/j.1525-1594.2005.29067.x 15854216

[15] Sayani R, Anwar M, Tanveer-ul-Haq, Al-Qamari N, Bilal MA Outcome of radiologically placed tunnelled haemodialysis catheters. J Coll Physicians Surg Pak. 2013;23(12):837–841. 24304984

[16] Gilmore J KDOQI clinical practice guidelines and clinical practice guideline for vascular access AJKD Submission Draft National Kidney Foundation [homepage on the Internet]; 2019 [cited 25 Sep 2019]. Available from: https://www.kidney.org/sites/default/files/kdoqi_vasc-access-review2019_v2.pdf

[17] Bisiwe F, Van Rensburg B, Barrett C, Van Rooyen C, Van Vuuren C Haemodialysis catheter-related bloodstream infections at Universitas Academic Hospital, Bloemfontein: Should we change our empiric antibiotics? S Afr J Infect Dis. 2015;30(1):29–33. 10.1080/23120053.2015.1103960

[18] Sampathkumar K, Ramakrishnan M, Sah AK, Sooraj Y, Mahaldhar A, Ajeshkumar R Tunneled central venous catheters: Experience from a single center. Indian J Nephrol. 2011;21(2):107–111. 10.4103/0971-4065.82133 21769173 PMC3132329

[19] Lacson E, Wang W, Lazarus JM, Hakim RM Change in vascular access and mortality in maintenance hemodialysis patients. Am J Kidney Dis. 2009;54(5):912–921. 10.1053/j.ajkd.2009.07.008 19748717

[20] Ethier J, Mendelssohn DC, Elder SJ, Hasegawa T, Akizawa T, Akiba T et al Vascular access use and outcomes: An international perspective from the dialysis outcomes and practice patterns study. Nephrol Dial Transplant. 2008;23(10):3219–3226. 10.1093/ndt/gfn261 18511606 PMC2542410

